# Physiotherapy students’ perspectives of online e-learning for interdisciplinary management of chronic health conditions: a qualitative study

**DOI:** 10.1186/s12909-016-0593-5

**Published:** 2016-02-16

**Authors:** Peter Gardner, Helen Slater, Joanne E. Jordan, Robyn E. Fary, Jason Chua, Andrew M. Briggs

**Affiliations:** School of Physiotherapy and Exercise Science, Curtin University, GPO Box U1987, Perth, WA 6845 Australia; HealthSense (Aust) Pty. Ltd., Melbourne, VIC 3204 Australia; Department of Health, Government of Western Australia, PO Box 8172, Perth Business Centre, Perth, WA 6849 Australia; Arthritis and Osteoporosis Victoria, PO Box 130, Caulfield South, VIC 3162 Australia

**Keywords:** Policy, Workforce, Physiotherapy, Rheumatoid arthritis, Digital, e-Learning, e-technology

## Abstract

**Background:**

To qualitatively explore physiotherapy students’ perceptions of online e-learning for chronic disease management using a previously developed, innovative and interactive, evidence-based, e-learning package: Rheumatoid Arthritis for Physiotherapists e-Learning (RAP-eL).

**Methods:**

Physiotherapy students participated in three focus groups in Perth, Western Australia. Purposive sampling was employed to ensure maximum heterogeneity across age, gender and educational background. To explore students’ perspectives on the advantages and disadvantages of online e-learning, ways to enhance e-learning, and information/learning gaps in relation to interdisciplinary management of chronic health conditions, a semi-structured interview schedule was developed. Verbatim transcripts were analysed using inductive methods within a grounded theory approach to derive key themes.

**Results:**

Twenty-three students (78 % female; 39 % with previous tertiary qualification) of mean (SD) age 23 (3.6) years participated. Students expressed a preference for a combination of both online e-learning and lecture-style learning formats for chronic disease management, citing flexibility to work at one’s own pace and time, and access to comprehensive information as advantages of e-learning learning. Personal interaction and ability to clarify information immediately were considered advantages of lecture-style formats. Perceived knowledge gaps included practical application of interdisciplinary approaches to chronic disease management and developing and implementing physiotherapy management plans for people with chronic health conditions.

**Conclusions:**

Physiotherapy students preferred multi-modal and blended formats for learning about chronic disease management. This study highlights the need for further development of practically-oriented knowledge and skills related to interdisciplinary care for people with chronic conditions among physiotherapy students. While RAP-eL focuses on rheumatoid arthritis, the principles of learning apply to the broader context of chronic disease management.

**Electronic supplementary material:**

The online version of this article (doi:10.1186/s12909-016-0593-5) contains supplementary material, which is available to authorized users.

## Background

### Health workforce capacity-building in chronic disease management

For a variety of macroeconomic and burden of disease factors (particularly musculoskeletal diseases [1]), a global mismatch exists between the health workforce supply and demand [2], highlighting a pressing need for capacity-building in the area of chronic disease management. Health policy, through Models of Care and whole-of-health frameworks also emphasises the need to address evidence-practice and burden-service gaps and to build capacity and resilience in the current and emerging health workforce in the area of chronic disease management [3–7].

### Education modes for the emerging workforce: the role of e-learning

Training the emerging health workforce requires sound pedagogical models that are innovative, engaging, cost effective, accessible, sustainable and evidence-based. In this regard, e-learning provides one mechanism, or *enabler*, for universities to effectively upscale capacity. E-learning can be defined as ‘…an approach to teaching and learning, representing all or part of the educational model applied, that is based on the use of electronic media and devices as tools for improving access to training, communication and interaction, and that facilitates the adoption of new ways of understanding and developing learning’ (p.92) [2]. More traditional education approaches include conventional lectures and face-to-face delivery. A recent systematic review of the use of online e-learning for undergraduate health professional education concluded that online e-learning is equivalent, or possibly superior, to traditional learning [8]. However, the variable quality of the studies included, and the significant risk of bias and study heterogeneity identified, all argue for more robust methodological designs and careful interpretation of the data in the interim.

### The role of the present study in the context of e-learning

Any evaluation of the effectiveness of online e-learning should consider students’ knowledge, skills, attitudes and satisfaction, in combination with an analysis of the potential advantages and disadvantages of e-learning [8]. This issue has largely remained unexplored in the context of the application of e-learning for the upskilling of the emerging health workforce in the management of chronic disease [9], despite recent recommendations that knowledge translation resources for physiotherapy should be in online format [10]. Although the use of e-learning in physiotherapy education has been explored previously [11–15], these studies have not specifically examined the role of e-learning in chronic disease management within an interprofessional context. Further, a recent non-randomised trial evaluated the efficacy of an interprofessional education program involving four disciplines including physiotherapy students [16]. In that study, e-learning was not used to deliver education and while students’ attitudes were measured using Likert scales, detailed exploration of their perceptions about the learning mode and experiences were not measured. Previously, we developed and evaluated an online e-learning package, Rheumatoid Arthritis for Physiotherapists e-Learning (RAP-eL) [17]. Data from our recent randomised controlled trial (RCT) using RAP-eL as an online e-learning intervention provides evidence for its effectiveness among the current practicing workforce [17]. One of the key advantages of RAP-eL is the use of real world clinical scenarios that help clinicians apply key principles of chronic disease management into knowledge and skills that translate into applied clinical practice. As an extension of this previous body of research, and in the context of scalability to the emerging workforce, we therefore sought to explore the implementation of RAP-eL into undergraduate education within the context of chronic disease management for physiotherapy students. Therefore, the aim of this study was to qualitatively explore students’ perceptions of the use of RAP-eL in a pre-licensure physiotherapy cohort.

## Methods

### Study design and research approach

To address the research aim, we used a qualitative study employing focus groups. The rationale underpinning the use of qualitative methods was to explore students’ perceptions of e-learning and gain insight into their behaviours, which is not readily accessible through surveys or other quantitative methods [18, 19]. This approach ensures meanings, experiences and views of participants are in their own words, rather than constraining to categories or terms imposed on them by others, which may be considered a limitation with purely quantitative approaches in recent literature [16]. As asserted by Cleary et al. [20], focus groups are the most appropriate format for exploring clinical and professional issues, because of the added advantage of interactivity and group discussion around the topic. Consistent with a qualitative approach, the number of focus groups conducted was based on the quality of data obtained to gain an in-depth understanding of students’ perceptions and views on e-learning. That is, not on the generalisability of the data nor on the sample size; a common misconception related to the conduct of qualitative research [21].

The study was nested as part of a broader research program examining outcomes of online e-learning for chronic disease management in health science students (using RAP-eL as the operational example). Focus groups were conducted using a semi-structured schedule developed and piloted by the multi-disciplinary project team. Consistent with the study aim, the schedule sought to explore: i) students’ perspectives on the advantages and disadvantages of online e-learning, and ii) ways to enhance e-learning, and identify information/learning gaps, in relation to interdisciplinary management of chronic health conditions. The manuscript is reported consistent with the COREQ-32 criteria (Additional file 1) [22].

### Focus group participants and sampling

Participants were drawn from physiotherapy students enrolled in either the third year of the Bachelor of Science (Physiotherapy) or second year of the Master of Physiotherapy (Graduate Entry) courses at Curtin University, Western Australia, and who had consented to participate in the broader program of research. Students were all enrolled in a unit of study that covered principles of chronic disease management. Students received three introductory lectures on the broad principles of chronic disease management before being given access to RAP-eL. Purposive demographic sampling of the students participating in the broader program of research was undertaken, in order to reflect heterogeneity across age, gender and educational background of the cohort. Members of the research team who were not directly known to the student group (HS, JC), invited, via email, those students purposively sampled to submit an expression of interest (EOI) to participate.

### Ethics, consent and permissions

Ethics approval for this study was received from the Human Research Ethics Committee at Curtin University, Australia (reference number PT001/2014). The study adhered to the Declaration of Helsinki. All participants in this study, provided informed consent, including consent to publish de-identified participant-level data.

### Online e-learning platform as the operational example

The RAP-eL package is designed for physiotherapists to increase their disease-related knowledge and clinical skills regarding the safe and effective management of rheumatoid arthritis (RA) within a chronic disease management framework [17]. The purpose of the resource is to deliver contemporary evidence for physiotherapy management of RA (*knowledge*), and to integrate that information with practice-relevant clinical skills (*skills*). The decision to build a modular-based, online e-learning package was reached through our initial research with clinicians regarding the mode of delivery they preferred. Overwhelmingly, clinicians nominated online e-learning as their preferred mode (78 %) [23]. Clinical content was identified in our previous study, which sought to define essential learning criteria by performing an international Delphi study and critical appraisal of 15 RA clinical guidelines [24], as well as areas of practice identified by clinicians as important in RA [24–26]. These data were further verified by examining other critically-appraised RA clinical guidelines, identified through a literature search [27, 28], and where evidence was lacking, expert opinion from a multidisciplinary advisory group.

RAP-eL was developed within an adapted knowledge-to-action framework [29] and uses a combination of problem-based learning and asynchronous learning, to strengthen clinical decision making and allow users to complete the training at their own pace, respectively. The knowledge-to-action conceptual framework comprises two distinct, but related components: (i) knowledge creation and (ii) the action cycle. The action cycle represents the activities needed for knowledge to be applied in practice [30]. Strategies recognised as effective in: facilitating evidence-based practice; changing practice behaviour and; acknowledging barriers to practice change [31, 32] were central to the design of RAP-eL. Strategies to lever active clinician engagement were embedded within learning modules with a focus on valid instruments for: screening and diagnosis; practical, usable evidence summaries; clinical skills demonstrations; modular-based learning; and consumer narratives [33, 34]. Our group has previously demonstrated, these approaches to be effective for Australian practitioners, in both urban and remote settings [35, 36]. RAP-eL can be accessed at http://www.rap-el.com.au.

### Data collection

Three face-to-face focus groups were conducted with students between September and October 2014 at Curtin University using the semi-structured schedule. Consistent with recommendations in the literature [20, 37], we determined *a priori* to conduct two focus groups consisting of more than four and less than 12 participants. The first two focus groups were conducted simultaneously in September. Data were analysed inductively after conducting these first two focus groups to identify themes. A third focus group was conducted in October, in order to verify the emergent themes and to determine whether data redundancy had been achieved. Here, redundancy refers to sequentially collecting data until all concepts are repeated multiple times without new concepts or themes emerging [38]. A senior physiotherapy educator (PG); and a senior physiotherapy educator/clinical researcher (HS) (both experienced in facilitating student focus groups) facilitated the focus groups. A secondary facilitator was also present at each focus group (JC, RF), and was responsible for observing and taking field notes. All focus groups were audio-recorded. Durations for the three focus groups ranged between 35 and 70 min.

### Data analysis

Focus group recordings were transcribed verbatim and transcripts were sent to all student participants for verification and no changes were requested. The qualitative data were analysed by a senior, independent researcher (JEJ). An inductive approach based on grounded theory was used to derive key themes and subthemes from the focus group transcripts until no new themes emerged [39]. The focus groups transcripts were analysed independently by a second researcher (AMB) to confirm the themes identified and discuss any discordance. Themes were refined to reach consensus, where necessary.

## Results

Twenty-three students (78 % female; 39 % with previous tertiary qualification) of mean (SD) age 23 (3.6) years, age range 19 to 33 years, participated. Twenty-one (91 %) students were sampled from the undergraduate course and two (9 %) from the graduate entry course, reflecting the usual proportions enrolled in each course. Forty-four per cent had previous experience with e-learning. Three key themes were identified:

1. Preference for a combination of online e-learning and lecture-style learning modes given the advantages and disadvantages of each.
2. Using ‘real world’ clinical examples to bridge the gap between theory and practice.
3. Strategies to facilitate consolidation of e-learning.

### Key theme 1: Preference for a combination of online e-learning and lecture-style learning modes given the advantages and disadvantages of each

While students perceived online e-learning as acceptable, blended delivery modes were considered important for the chronic disease management curriculum. Students were cognisant of the advantages and disadvantages of both online e-learning and lecture-based learning modes. Flexibility to work and learn at one’s own pace with ready and repeatable access to comprehensive information were key perceived benefits of online e-learning learning compared to a lecture mode.

“*I think online also you kind of get a more in-depth amount of information because you can read it yourself at your own time rather than having to fit a certain amount of information into like a one-hour, two-hour lecture. You can spend like half an hour blocks trying to get that information. So it’s all* [set] *out there for you and it’s really well explained, whereas someone in a lecture has only a certain amount of time to kind of go over it…and with less detail than what you can get online*” [P16]

Ease of access to comprehensive information e-learning was also valued by students who indicated that they were likely to use e-learning resources throughout their learning.

“*So just ease of having an electronic-based and nice, like, document to refer back to in future cases and what not*” [P19]

Whereas traditional lecture-style education usually requires students to physically attend an education session, disadvantages reported about the e-learning experience included: the need to be personally motivated to complete learning modules; and the risk of “combing for answers” to quizzes instead of active, comprehensive learning of information.

“*…if I had a lot of these to do* [e-learning learning packages] *then I would very likely procrastinate or skim read through them or not pay as much attention because it’s not – I’m not necessarily having someone there. I think the face-to-face lectures they kind of make me go because it’s like you have to be there at this time and they’ll do it so I’ll turn up, whereas if it’s online then you’re like, “Oh, yeah, I might do it later or do it at this time,” and it just keeps getting put off.*” [P21]

“…*sometimes I try to just find the answer rather than just understanding. For example, question is there, so I try to just pick up the answer rather than understanding more.*” [P3]

Students reported valuing particular attributes of face-to-face learning, including the ability to seek clarity on topics as information was being delivered. Students also appreciated verbal cues, such as tone of voice, to emphasise the importance of key concepts.

*“I guess if it’s face to face with the lecture, like if you have question, like, along the way you can always pop it up. After that you still have to go to a process of emailing and stuff and I end up being lazy and just, oh, it’s okay; it’s just a small concept issue. And you might just miss the understanding part of it*” [P5]

Students identified that a combination of online e-learning and lecture learning modes would be beneficial for learning about chronic disease management. Specifically, they cited a preference for an adjunctive lecture or tutorial after completion of e-learning materials to ask questions and seek clarifications, and it was perceived that e-learning would only be suitable for select units.

“*Personally, like, I really wanted to do a tutorial at the end of it* [RAP-eL] *with a face to face lecture where I could nut out things that I didn’t quite get that I couldn’t pick up from that*.” [P8]

*“… chronic diseases probably is a good one to have as like an online package but, yeah, probably other units…wouldn’t suit online packages as well*.” [P14]

### Key theme 2: Using ‘real world’ clinical examples to bridge the gap between theory and practice

Integrated, clinically-oriented interdisciplinary learning that focuses on knowledge and skills to encourage “learning through doing” is an important attribute of online e-learning, as perceived by physiotherapy students for chronic disease management. Within e-learning resources, students expressed the need for greater insights into practical, clinical behaviours appropriate for physiotherapy consultations involving a person with a chronic disease. Specifically, students identified the importance of practical examples demonstrating how to develop a physiotherapy management plan and different consultation approaches for patients with chronic health conditions. There was particular interest in viewing videos of physiotherapy consultations and having interactive case studies where students could see and then practice developing a treatment plan and then review best-practice examples.

“ *…I know when it comes to like management and treatment, it is individual based but maybe just more of like a specific kind of direction on exactly what kind of things you do… I know there’s like the basic kind of information on what you would do but maybe if it was just expanded more so that we could actually have better understanding on…, what a treatment session would be or what kind of home exercises or management plan you would give them…*” [P14]

“*Maybe a video on an actual treatment session, so that way we can see how like the physio goes about treating the patient and then, especially if it’s an actual patient with RA, then we can see how we need to approach them and …what’s appropriate*.” [P15]

Additionally, students clearly expressed uncertainty in their knowledge and skills surrounding the use of interprofessional practice as it related to the management of chronic disease in the broader physiotherapy curriculum. There was clear support for the use of more explicit, practical examples of interprofessional practice to increase their confidence in clinical situations. Specifically, practically-orientated materials that detail the roles of different health professionals and how to identify which health professional to refer a patient to, when it is appropriate to refer, and how to refer were considered important:

“…*there was a section on what the rheumatoid specialist actually does…if you actually have that for an OT [Occupational Therapist], a psychologist, a nutritionist … maybe just a paragraph with just a couple of bullet points and this is how their stuff integrates in as well, it would paint a better picture…It would be good just to have more of an idea of the holistic approach*…” [P13]

### Key theme 3: Strategies to facilitate consolidation of e-learning

Students were unequivocal in their suggestions to optimise the e-learning experience. Quizzes at the end of each e-learning module were identified as a critical tool to assist students to consolidate their learning, integrate information and translate it into a clinical context, and also to provide feedback on learning progression. Additionally, students indicated that imposing time limits would assist timely completion of each e-learning RAP-eL module.

“*I think having quizzes or something like that is a good thing as part of the package because it puts it into a case study or it makes you sort of consolidate your knowledge. So I think it’s important to have that at the end, but then also getting feedback on it. A lot of our assessments throughout the semester we don’t actually get any feedback and it’s really frustrating because none of us actually improve anywhere*” [P18]

Including a ‘frequently asked questions’ section and/or an e-learning discussion blog or forum with experts or experienced peers who could provide guidance in relation to treatment practices were identified as additional strategies to facilitate learning.

*“If they had something like frequently asked questions maybe they can refer back to that, so they can go, “Yes, people have been asking questions. They will actually answer me on this.” So that might actually be useful, a page like that*” [P4]

Further, students indicated that learning may be enhanced using the blogging and social networking technologies across student disciplines; that is, essentially creating a ‘virtual learning community’. As part of a discussion blog or forum it was suggested that it would be beneficial to have access to multidisciplinary health professionals to reduce the gap between theory and practice for an interdisciplinary approach to chronic disease management.

“*…like we were talking before about the OT…we would then be able to still communicate with an OT and be like, “What would you recommend?” or a dietician or something like this, “What information can I give in the interim?”* [P6]

## Discussion

To our knowledge, this is the first study to investigate the acceptability among physiotherapy students of e-learning, as it relates to pre-licensure knowledge acquisition and perceived confidence in skills for the interdisciplinary management of a chronic health conditions. Physiotherapy students in our study found online e-learning for chronic disease management to be acceptable.

### Blended learning styles

While tertiary institutions may see multi-level value (scalability, cost effectiveness, mitigation of access and temporal barriers) in transitioning educational content to e-learning modes, particularly in light of emerging evidence for the effectiveness and acceptability of this mode in both online [2, 8, 40] and offline formats [41], the advantages of blended learning models remain [2, 8]. In this context, a key to achieving successful educational outcomes appears to be the integration of technology with sound pedagogy and practice-relevant content knowledge [42]. A mix of delivery styles that best align with the requirements of work-force ready health professionals is indicated [43]. This is consistent with our findings where, rather than indicating a preference for one format over another, students felt that a blended mode added value in different ways to the learning experience in motivating and developing them in learning about chronic disease management. The preference for a blended learning style is consistent with previous research among health sciences students [44]. Further, recent systematic reviews suggest that blended approaches to learning are no worse than more traditional forms of educational instruction, and in some cases better, when evaluating student competence post-instruction [2, 41, 45]. Given the requirement for both knowledge and skills-based competencies, set against the real world of complex clinical decision making [46], the use of blended delivery is appropriate for pre-licensure health professional training. In our study, students perceived that some content, especially some practical physiotherapy skills, could not be effectively delivered online, in contrast to evidence from a recent non-randomised controlled trial suggesting that online delivery of practical physiotherapy content is both feasible and effective compared with traditional teaching modes [15].

### Optimising e-learning for interdisciplinary management of chronic health conditions

Grounding the theory of management of chronic conditions such as RA, in practical, clinically-relevant examples, was a clearly identified theme. This correlates strongly with principles of adult learning, where learners’ reactions to educational resources are more positive when they see a direct connection to their current or future practice [47, 48]. For the emerging workforce, it may also reflect a lack of experiential learning, whereas practicing clinicians have a more developed skillset in translating knowledge into practice [17, 35, 36]. Educators need to be aware of the need for e-learning resources to explicitly translate content knowledge (i.e. ‘*knowing*’) into skills-based competencies using clinically-relevant scenarios (i.e. ‘*doing*’) [26, 29, 35, 36]. The importance of bridging this ‘know-do gap’ is not just limited to student learning. For example, in our recent RCT of practicing clinicians, we identified at 8 weeks follow-up that knowledge was retained to a greater extent than confidence in clinical skills for best-practice management of RA [17]. As recommended in recent reviews [2, 8, 45], interactive and downloadable resources are readily updatable, and allow for iterations and alignment with changes in curriculum to best reflect interprofessional learning.

Students also identified other strategies to further assist with learning, including discussion blogs/fora and quick quizzes that targeted knowledge and skills. The effectiveness of blogging and social networking as tools to enhance clinical reasoning and metacognition among physiotherapy students have also been identified [11, 14]. While these interactive strategies may well be appealing and facilitate learning, their implementation needs to be pragmatically balanced against the resourcing requirements needed for adequate moderation and maintenance. This is particularly relevant when online e-learning resources are delivered at scale across multiple institutions and countries, as we have undertaken with RAP-eL. Blogs have similarly been identified as useful among nursing students, although the perceived effectiveness of this technology in that cohort was varied and less favoured by students who were more familiar with face-to-face education, compared with distance education students [49]. A similar finding was reported by Peacock and Hooper in 2007 [13] among physiotherapy students, although this may relate to attitudes towards blogging technologies almost one decade ago compared to more contemporary student perspectives on this issue.

### Interprofessional practice for chronic disease management

Interprofessional models of health education for chronic disease management identify domain-specific knowledge and skills, as well as generic, cross-discipline skills, to be essential [24, 50]. Students in our study identified that online discipline-specific student education, while delivered in an interprofessional framework, needs to explicitly demonstrate discipline-specific and interdisciplinary practices that resonate with real world contexts. Consistent with previous literature [51, 52], we identified that students perceived a disconnect between theory and practical skills in interdisciplinary practice related to chronic disease management and saw value in e-learning packages being able to deliver practical information about the roles of different health professionals. In this context, we designed RAP-eL using an adapted knowledge-to-action framework [29] embedded with change strategies that are recognised as effective in: facilitating evidence-based practice; changing practice behaviour and; acknowledging barriers to practice change [31, 32]. This framework, and the embedded interdisciplinary content, allow for generalizability of the curriculum to other health training programs. In fact, we have recently collaborated to implement and explore the suitability and effectiveness of RAP-eL for upskilling third-year medical students, with data supporting effective implementation and upskilling [53]. These findings are also consistent with recent Australian data demonstrating the effectiveness of an online interprofessional module for cardiovascular disease management [12] and findings that online learning can improve students’ attitudes about working inter-professionally [54].

### Strengths and limitations

Our findings contribute to the literature investigating the adoption of online e-learning as a mechanism for upskilling the emerging health workforce. Our study focussed specifically on the acceptability of this mode of learning, consistent with research recommendations [8]. While recent systematic reviews have comprehensively evaluated the comparative effectiveness of specific e-learning applications compared with, or incorporating, other learning modes [8, 41], less discipline-specific research has been undertaken to explore users’ experiences of these modes of learning in contemporary university education. Importantly, the study design was grounded on an operational online e-learning example, RAP-eL. This approach enabled the student participants to discuss their perspectives from a concrete, experiential perspective rather than a theoretic frame of reference. Our findings are transferable as our sampling approach reflected both the demographic characteristics and usual proportions of students enrolled in the undergraduate and graduate-entry courses. Broad discussion, interaction and exchange of perspectives among students was facilitated by the use of focus groups [55]. This approach was consistent with earlier research in the area [12, 13, 49, 56], and may not have occurred with the use of individual interviews. However, because our study used only focus groups for data collection, some students may not have expressed all their views in a group setting. Finally, our study design was cross-sectional, and as such we are unable to speculate on the experiences of students’ learning over time, representing an important design consideration for future research in this area. Although methodological quality of qualitative research is somewhat related to adequacy of sample size [18, 56], the rigour of the research design is more dependent on a range of other methodologic components [21]. Our study has been appropriately conducted as evidenced our methods. First, we employed a demographic purposive sampling approach [57] ensuring spread across age, gender, educational background and course with proportions reflective of the total student cohort. Second, our data were analysed inductively using a grounded theory approach. Third, we achieved redundancy in data which was confirmed by undertaking data analysis after the first two simultaneously conducted focus groups were completed and then verifying the emergent themes in a third focus group.

## Conclusions

Blended learning modes that resonate with real world, integrated clinical practice were preferred as a mechanism for upskilling the emerging physiotherapy health workforce within an interdisciplinary framework. Students articulated that e-learning platforms should ideally demonstrate practical, discipline-specific and cross-discipline practices as they relate to care delivery for people with chronic health conditions. Students identified that e-learning can be enhanced for the user when specific tools are embedded in a platform, including quizzes and clinically-oriented blogs.

## Additional file

Additional file 1:
**COREQ-32 checklist**. (DOCX 111 kb)

## Abbreviations

RAP-eL: Rheumatoid arthritis for physiotherapists – eLearning
RCT: Randomised controlled trial
