# Supplementary material for: Physiotherapy students’ perspectives of online e-learning for interdisciplinary management of chronic health conditions: a qualitative study
Source: BMC Med Educ. 2016 Feb 16;16:62. doi: 10.1186/s12909-016-0593-5 (PMC4754862; doi:10.1186/s12909-016-0593-5)
Supplement: Additional file 1: — COREQ-32 checklist. (DOCX 111 kb) [file 12909_2016_593_MOESM1_ESM.docx]

Consolidated criteria for reporting qualitative studies (COREQ):

32-item checklist [1].

| **No. Item** | **Guide questions/description** | **Addressed in manuscript / response** |
| --- | --- | --- |
| **Domain 1: Research team and reflexivity** | | |
| *Personal Characteristics* |  |  |
| 1. Inter viewer/facilitator | Which author/s conducted the inter view or focus group? | Reported in “Data collection” |
| 2. Credentials | What were the researcher’s credentials? E.g. PhD, MD | No. Not required by journal |
| 3. Occupation | What was their occupation at the time of the study? | Reported in “Data collection” |
| 4. Gender | Was the researcher male or female? | Reported on Title Page |
| 5. Experience and training | What experience or training did the researcher have? | Reported in “Data collection” |
| *Relationship with participants* |  |  |
| 6. Relationship established | Was a relationship established prior to study commencement? | Reported in “Data collection” |
| 7. Participant knowledge of the interviewer | What did the participants know about the researcher? e.g. personal goals, reasons for doing the research | All participants were provided with a detailed Participant Information Statement that outlined the background to the study, aims, and requirements for participation. |
| 8. Interviewer characteristics | What characteristics were reported about the inter viewer/facilitator? e.g.  Bias, assumptions, reasons and interests in the research topic | Reported in “Data collection” |
| **Domain 2: study design** | | |
| *Theoretical framework* |  |  |
| 9. Methodological orientation and Theory | What methodological orientation was stated to underpin the study? e.g.  grounded theory, discourse analysis, ethnography, phenomenology, content analysis | Reported in “Data analysis” |
| *Participant selection* |  |  |
| 10. Sampling | How were participants selected? e.g. purposive, convenience, consecutive,  snowball | Reported in “*Focus group participants and sampling*” |
| 11. Method of approach | How were participants approached? e.g. face-to-face, telephone, mail, email | Reported in “*Focus group participants and sampling*” |
| 12. Sample size | How many participants were in the study? | 23 |
| 13. Non-participation | How many people refused to participate or dropped out? Reasons? | Zero. |
| *Setting* |  |  |
| 14. Setting of data collection | Where was the data collected? e.g. home, clinic, workplace | Reported in “Data collection” |
| 15. Presence of nonparticipants | Was anyone else present besides the participants and researchers? | No |
| 16. Description of sample | What are the important characteristics of the sample? e.g. demographic data, date | Reported in “Results” |
| *Data collection* |  |  |
| 17. Interview guide | Were questions, prompts, guides provided by the authors? Was it pilot tested? | Reported in “Study Design” |
| 18. Repeat interviews | Were repeat inter views carried out? If yes, how many? | No |
| 19. Audio/visual recording | Did the research use audio or visual recording to collect the data? | Reported in “Data collection” |
| 20. Field notes | Were field notes made during and/or after the inter view or focus group? | Yes |
| 21. Duration | What was the duration of the inter views or focus group? | Reported in “Data collection” |
| 22. Data saturation | Was data saturation discussed? | Discussed in “Data collection section of Methods and Strengths and limitations section of Discussion” |
| 23. Transcripts returned | Were transcripts returned to participants for comment and/or correction? | Yes |
| **Domain 3: analysis and findings** | | |
| *Data analysis* |  |  |
| 24. Number of data coders | How many data coders coded the data? | Reported in “Data analysis” |
| 25. Description of the coding tree | Did authors provide a description of the coding tree? | No |
| 26. Derivation of themes | Were themes identified in advance or derived from the data? | Themes were derived inductively from the data. Reported in “Data analysis” |
| 27. Software | What software, if applicable, was used to manage the data? | No |
| 28. Participant checking | Did participants provide feedback on the findings? | No. |
| *Reporting* |  |  |
| 29. Quotations presented | Were participant quotations presented to illustrate the themes/findings? Was each  quotation identified? e.g. participant number | Yes. Reported in Results |
| 30. Data and findings consistent | Was there consistency between the data presented and the findings? | Yes |
| 31. Clarity of major themes | Were major themes clearly presented in the findings? | Yes |
| 32. Clarity of minor themes | Is there a description of diverse cases or discussion of minor themes? | Yes |

1. Tong A, Sainsbury P, Craig J. Consolidated criteria for reporting qualitative research (COREQ): a 32-item checklist for interviews and focus groups. International journal for quality in health care : journal of the International Society for Quality in Health Care. 2007;19(6):349-57.
